# Comparative Endosymbiont Community Structures of Nonviruliferous and Rice Stripe Virus-Viruliferous *Laodelphax striatellus* (Hemiptera: Delphacidae) in Korea

**DOI:** 10.3390/v17081074

**Published:** 2025-08-01

**Authors:** Jiho Jeon, Minhyeok Kwon, Bong Choon Lee, Eui-Joon Kil

**Affiliations:** 1 Department of Plant Medicals, Gyeongkuk National University, Andong 36729, Republic of Korea; wjswlgh15@naver.com (J.J.); ysx0007@naver.com (M.K.); 2 Crop Protection Division, National Institute of Agriculture Sciences, Rural Development Administration, Wanju 55365, Republic of Korea; leebc21@korea.kr

**Keywords:** endosymbiont, rice stripe virus, *Laodelphax striatellus*, *Wolbachia*, microbial diversity

## Abstract

Insects and their bacterial endosymbionts form intricate ecological relationships, yet their role in host–pathogen interactions are not fully elucidated. The small brown planthopper (*Laodelphax striatellus*), a polyphagous pest of cereal crops, acts as a key vector for rice stripe virus (RSV), a significant threat to rice production. This study aimed to compare the endosymbiont community structures of nonviruliferous and RSV-viruliferous *L. striatellus* populations using 16S rRNA gene sequencing with high-throughput sequencing technology. *Wolbachia* was highly dominant in both groups; however, the prevalence of other endosymbionts, specifically *Rickettsia* and *Burkholderia*, differed markedly depending on RSV infection. Comprehensive microbial diversity and composition analyses revealed distinct community structures between nonviruliferous and RSV-viruliferous populations, highlighting potential interactions and implications for vector competence and virus transmission dynamics. These findings contribute to understanding virus-insect-endosymbiont dynamics and could inform strategies to mitigate viral spread by targeting symbiotic bacteria.

## 1. Introduction

Rice stripe virus (RSV: *Tenuivirus oryzaclavatae*) was first reported in Japan in the 1930s and is now known to be widespread in many countries, including Korea, Japan, and China [1,2]. RSV belongs to the genus *Tenuivirus* and has been assigned to the family *Phenuiviridae* in the order Bunyavirales [3]. The negative/ambisense RNA genome of RSV consists of four single-stranded (ss) RNA segments [4], and it can infect various plants and crops, such as corn, wheat, oats, and other weeds in addition to rice [5,6]. RSV is persistently transmitted by small brown planthoppers (SBPH, *Laodelphax striatellus*) [5]. RSV is acquired by SBPH through sap-sucking, spreading internally to the midgut, salivary glands, and ovaries, resulting in persistent infection and transovarial transmission through eggs [7].

SBPH, which has a significant impact on the transmission of RSVs, is one of the pests affecting various crops. SBPH is known to be prevalent in Palearctic regions such as China, Japan, Germany, Italy, Russia, and Kazakhstan [8]. SBPH is known to damage rice, maize, oat, wheat and barley through sap-sucking, and it is known as a vector insect that spreads viruses such as RSV, rice black-streaked dwarf virus (RBSDV, *Fijivirus alporyzae*; genus *Fijivirus*), and rice dwarf virus (RDV, *Phytoreovirus alphaoryzae*; genus *Phytoreovirus*) [5].

Various living beings, including humans, insects, and plants, harbor a diverse assortment of microorganisms collectively termed the “microbiota”. The compilation of microbial genetic material within a host is termed the “microbiome” [9]. These communities of microorganisms engage in interactions with their hosts, augmenting the ability of organisms to adapt to fluctuations in environmental conditions [10,11,12,13,14].

Among these symbionts, bacteria play diverse roles and have been extensively studied [15]. Insect endosymbionts actively engage in various facets of host life cycles, exerting profound influences on the biological characteristics of their insect hosts [16,17,18]. They also play pivotal roles in nutritional provision and fortifying the gut against colonization by exogenous species, including pathogens that could affect the respective vectors [12]. Consequently, there has been a surge in research interest surrounding insect symbionts in recent years [19]. A plethora of studies has underscored the involvement of endosymbionts in facilitating pathogen transmission within insect host vectors [20,21,22,23]. Notably, certain endosymbionts aid in pathogen entry into insects. For instance, GroEL proteins from various endosymbionts play a pivotal role in virus transmission in aphids and whiteflies by binding to virus particles, thus shielding them from rapid proteolytic degradation in the insect hemolymph [23,24,25,26].

Sequencing of the 16S ribosomal RNA (rRNA) gene has been widely employed as the primary method for bacterial identification [27,28]. The approximately 1500 base pairs of the 16S rDNA sequence provide ample material for bioinformatics analyses [29], and it is universally present across bacterial species with a well-defined function [30]. However, this approach encounters challenges in polymicrobial samples, where the presence of multiple bacterial species results in ambiguous Sanger sequencing results [31]. Next-generation sequencing (NGS) technologies have revolutionized high-throughput functional genomic research [32]. Notably, Illumina technology generates millions of DNA sequence reads in a single run, significantly altering the landscape of genetic studies [33]. Currently, NGS technologies offer novel avenues for analyzing the structure and content of microbial genomes [34], with applications spanning the investigation of microbial communities associated with insects such as beetles [35], ants [36], thrips [37], and planthoppers [38,39].

Despite advances in these analytical methods, limited studies have investigated the interactions between endosymbionts of SBPH and RSV, despite their significant impact on rice yield. Therefore, in this study, we used Illumina 16S rRNA gene MiSeq sequencing to observe changes in the endosymbiont of virus-free and RSV-viruliferous SBPH in Korea. This study explores the impact of RSV on the composition of the endosymbiont of SBPH and aims to provide a broader perspective for the control of RSV.

## 2. Results

### 2.1. Differences in the Diversity of SBPH with or Without RSV Infection

We utilized Illumina MiSeq to investigate SBPH to analyze bacterial microbiota diversity between virus-free SBPH (VF) and RSV-viruliferous SBPH (RSV). After filtering out low-quality reads, 241,586–327,278 useful reads were obtained from the samples. Operational taxonomic unit (OTU) clustering was performed using the SILVA 16s database, and a total of 61 OTUs were identified in both groups (VF, RSV). At a similarity cutoff of 99% (1% dissimilarity), 54 OTUs were identified in the VF group, whereas 27 OTUs were identified in the RSV-viruliferous group. In the two groups, the Shannon entropy, phylogenetic diversity, and Simpson’s index were 0.62 and 0.63; 1.59 and 1.11; 0.15 and 0.18, respectively (Table 1). Shannon entropy measures the diversity and evenness of an ecosystem. Phylogenetic diversity evaluates biodiversity based on the evolutionary distance among species. Simpson’s index reflects the dominance of particular species, where higher values indicate lower evenness and a greater presence of dominant species. Shannon entropy and Simpson’s index. Although Shannon entropy and Simpson’s index values were similar between the two groups, the phylogenetic diversity notably differed. Specifically, the virus-free SBPH population exhibited higher phylogenetic diversity, suggesting the presence of a broader range of bacterial lineages compared to the RSV-viruliferous populations. The ASV analysis also used the SILVA 16s database as a reference database. In the ASV analysis, Shannon entropy and Simpson’s index values were consistent with the OTU analysis, again showing similar diversity and evenness between the two groups. Additionally, phylogenetic diversity followed the same pattern observed in OTU analysis, reinforcing the conclusion of reduced bacterial diversity in RSV-viruliferous SBPH. However, the number of microbial communities identified was different between the OTU and ASV.

### 2.2. Bacterial Community Composition

**Figure 1:**
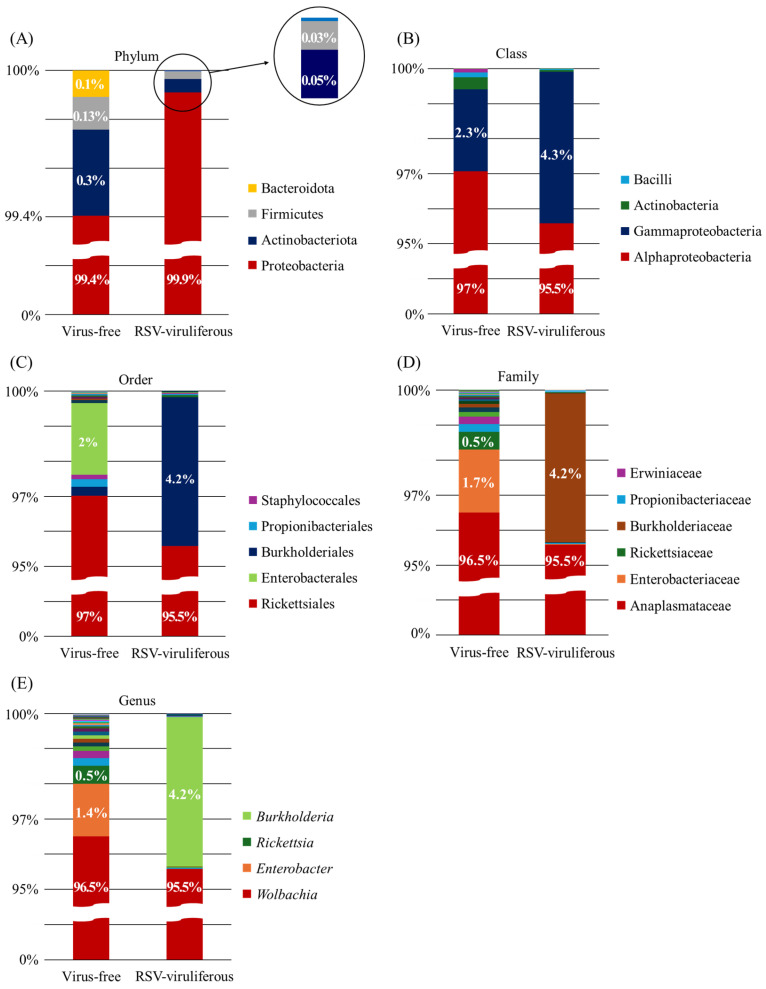
Bacterial community composition of virus-free and RSV-viruliferous SBPH populations based on OTU analysis. (**A**) Phylum-level: Proteobacteria was dominant in both groups. (**B**) Class-level: Alphaproteobacteria dominated virus-free (97%) and RSV-viruliferous (95.5%) groups; Gammaproteobacteria was present in lower abundances (2.3% virus-free; 4.3% RSV-viruliferous). (**C**) Order-level: Rickettsiales was predominant; smaller proportions of Burkholderiales and Enterobacterales were also detected. (**D**) Family-level: Anaplasmataceae dominated both groups, with minor representation from Burkholderiaceae and Enterobacteriaceae. (**E**) Genus-level: *Wolbachia* dominated in both groups, while *Rickettsia* and *Enterobacter* were present only in virus-free samples, and *Burkholderia* was notably observed in RSV-viruliferous samples. Percentages indicate relative abundance at each taxonomic level.

To evaluate the OTUs distribution among different SBPH populations, we identified 20 OTUs that were common to both populations. This indicates that more than half of the endosymbionts in the RSV group belonged to the shared portion, with most OTUs classified under the genus *Wolbachia*. Further, the number of unique OTUs was 34 and 7 in the VF and RSV populations, respectively, which were represented by a low proportion (Table S1). Comparing the two groups, at least four phyla were identified in each sample at the phylum level (Figure 1A). *Proteobacteria* comprised the majority of all samples, with 99.4% in the VF group and 99.9% in the RSV group, respectively. Further analysis was performed to show the relative bacterial abundances at the class, order, family and genus levels (Figure 1B–E). At the class level, Alphaproteobacteria dominated in both groups, accounting for 97% in the virus-free (non-viruliferous) group and 95.5% in the RSV-viruliferous group, with smaller proportions of Gammaproteobacteria (2.3% and 4.3%, respectively) (Figure 1B). At the family level, apart from Anaplasmataceae, which accounted for 96.5% in the Virus-free group, Enterobacteriaceae made up 1.7%. In the RSV-viruliferous group, besides Anaplasmataceae, which accounted for 95.5%, Burkholderiaceae represented 4.2% (Figure 1D). At the genus level, a total of 35 genera were classified across both groups, with 12 genera common to all SBPH groups. Additionally, 21 genera were unique to the VF group, and 2 unique genera were identified in the RSV group. *Wolbachia* was dominant in both the VF group (96.5%) and the RSV group (95.5%). Other genera showed lower percentage, with *Enterobacter* and *Rickettsia* comprising 1.4% and 0.5%, respectively, in the VF group, and *Burkholderia* accounting for 4.2% in the RSV group (Figure 1E and Figure S1). This fact demonstrate that the bacterial distribution is predominantly dominated by the genus *Wolbachia*, with some variations depending on RSV infection.

**Figure 2:**
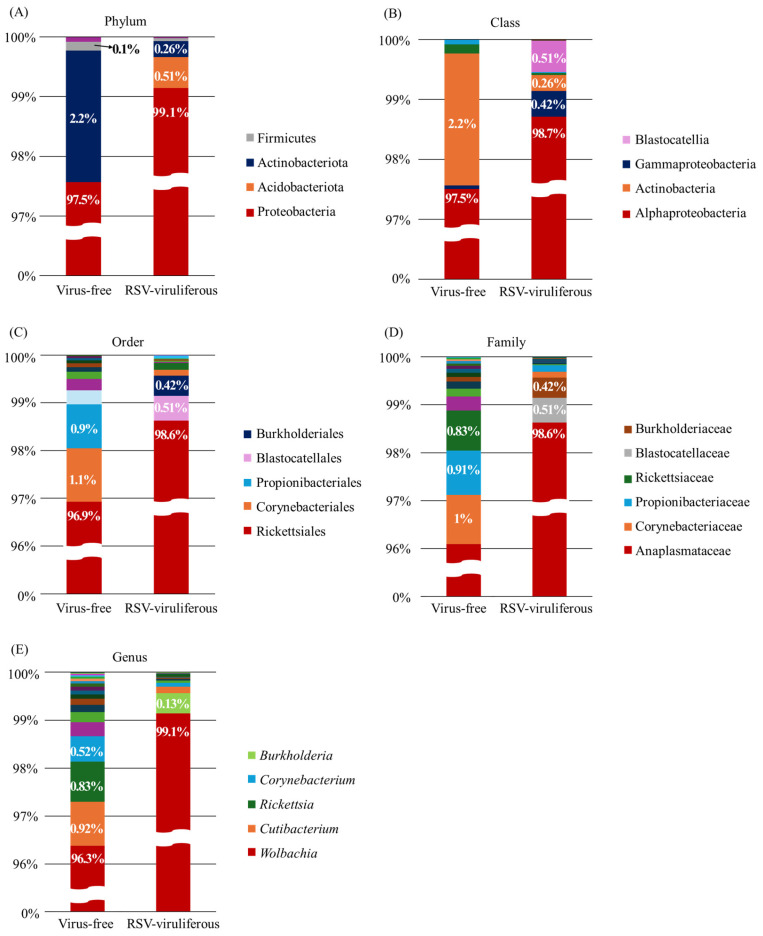
Bacterial community composition of virus-free and RSV-viruliferous SBPH populations based on ASV analysis. (**A**) Phylum-level: Proteobacteria dominated both groups. (**B**) Class-level: Alphaproteobacteria were predominant (97.5% virus-free; 98.7% RSV-viruliferous), accompanied by small proportions of other classes. (**C**) Order-level: Rickettsiales was dominant, with minor occurrences of Corynebacteriales, Propionibacteriales, Blastocatellales, and Burkholderiales. (**D**) Family-level: Anaplasmataceae dominated in both groups, with minor contributions from Corynebacteriaceae, Propionibacteriaceae, Rickettsiaceae, Blastocatellaceae, and Burkholderiaceae. (**E**) Genus-level: *Wolbachia* dominated both groups; small proportions of *Rickettsia*, *Cutibacterium*, and *Corynebacterium* were identified in virus-free samples, whereas *Burkholderia* was minimally detected (0.13%) in RSV-viruliferous samples. Percentages indicate relative abundance at each taxonomic level.

The ASV analysis identified seven ASVs common to both populations. Consistent with the OTU analysis, the majority of the ASVs were classified in the genus *Wolbachia*, with a low percentage of unique ASVs (17 and 10, respectively). Comparing the two groups, at least four phyla were identified at the phylum level (Figure 2A). In both groups, Proteobacteria comprised the majority of the samples at 97.5% and 99.1%, respectively. Further analysis organized the relative abundance of bacteria at the class, order, family, and genus levels (Figure 2B–E). At the class level, Alphaproteobacte-ria was dominant in both groups, with 97.5% in the virus-free (non-viruliferous) group and 98.7% in the RSV-viruliferous group, slightly higher in the RSV-viruliferous group, contrary to the OTU analysis (Figure 2B). At the family level, Corynebacteriaceae, Propionibacteriaceae, and Rickettsiaceae comprised the majority of the virus-free group, except for Anaplasmataceae, which accounted for 96% and 98.6%, respectively, while Blastocatellaceae and Burkholderiaceae comprised the remainder of the RSV-viruliferous group (Figure 2D). At the genus level, bacterial community composition trends were largely consistent between OTU and ASV analyses. However, notable differences included the absence of *Enterobacter* in the virus-free group in ASV analysis, which was present in OTU analysis, and a significant reduction in the abundance of *Burkholderia* from 4.2% (OTU) to 0.13% (ASV) in the RSV-viruliferous group. These discrepancies highlight the methodological sensitivity and underscore the importance of integrating multiple analytical approaches.

### 2.3. Validation of Sequencing Data

To validate our sequencing results, PCR was performed to specifically detect and confirm the presence of selected endosymbionts, particularly targeting genera (*Rickettsia* and *Burkholderia*) whose relative abundances varied significantly between groups, excluding the highly dominant genus *Wolbachia* (validation shown in Figure S1). The PCR validation results were fully consistent with our sequencing data. Specifically, amplification of *Rickettsia* DNA was successful only in virus-free SBPH samples, while *Burkholderia* DNA was exclusively amplified in RSV-viruliferous SBPH samples, clearly confirming the sequencing-based findings.

## 3. Discussion

In this study, 16S rRNA sequencing was used to identify changes in the endosymbiont of SBPH according to RSV infection. The most common clustering method in metagenomic studies is identity clustering, which uses a fixed sequence identity criterion (usually 97% for sequences from the same species) to generate operational taxonomic units (OTUs) [40]. In recent years, denoising has been introduced as an alternative clustering method that forms clusters through a process of predicting and correcting for noise. This method is called amplicon sequence variants (ASVs) [41,42,43] and forms clusters after correcting for true sequencing error (noise). Denoising approaches utilize well-established statistical models to identify low-frequency sequences and consider them as sequence variants. This produces fewer clusters, but with greater consistency and precision between clusters and thoroughly validated results [44,45,46]. Recently, ASVs methods have been increasingly used in microbiota research alongside traditional OTUs methods. Employing both OTU and ASV analyses allowed us not only to cross-validate our results but also to capture both coarse-grained (OTU) and fine-grained (ASV) insights into the endosymbiont community composition. Although general patterns were consistent, differences highlighted by ASV analysis provide additional resolution, enabling a deeper understanding of microbial dynamics.

In this study, we utilized both OTU and ASV analyses to characterize bacterial communities and identified *Wolbachia* as the overwhelmingly dominant endosymbiont, comprising 96.5% in the VF group and 95.5% in the RSV group. This result aligns well with previous reports highlighting *Wolbachia*’s prevalence in SBPH [47]. *Wolbachia* is a widely distributed endosymbiont belonging to the Alphaproteobacteria, known to infect numerous hosts including insects, isopods, and spiders [48,49,50,51]. *Wolbachia* are maternally transmitted and have evolved several strategies to facilitate their own proliferation and transmission. These include parthenogenesis, feminization, male killing, and cytoplasmic incompatibility [52,53,54,55,56,57], and these strategies regulate the reproduction of their hosts. Teixeira et al. [58] reported that *Wolbachia* infection increased *D. melanogaster*’s resistance to RNA viruses (e.g., Drosophila C virus, Nora virus and Flock house virus), reducing the viral load of infected flies. When *Wolbachia* was introduced into the mosquito *Aedes aegypti*, it induced resistance to Dengue virus and interfered with virus transmission [59]. *Wolbachia* strains from various insects transferred to *A. aegypt* have limited the replication of arboviruses such as dengue virus (DNEV), chikungunya virus (CHIKV), yellow fever virus (YFV), and zika virus (ZIKV) [60]. In addition, *Wolbachia* regulate the reproduction of insects by enhancing their gene transmission through the female germline. The most common variation is referred to as cytoplasmic incompatibility (CI).

In SBPH, *Wolbachia* infection is widely distributed in the head, thorax, abdomen, salivary gland, gut, ovary, and testis [61]. *Wolbachia* was able to induce strong CI in SBPH, which was one of the main features of the symbiosis between *Wolbachia* and SBPH [62,63]. CI occurs when eggs laid by *Wolbachia*-free (W-) females fail to develop after mating with *Wolbachia*-infected (W+) males. In contrast, the other three mating combinations (W+ female × W- male, W+ female × W+ male, and W- female × W- male) result in normal offspring development [48,64,65]. In addition, Zhang et al. [66] used 454-FLX pyrosequencing to show that the *Wolbachia* transcriptome in non-viral SBPH is four times larger than in RSV-viruliferous insects [48,64,65]. Zhang et al. [66] identified several *Wolbachia* genes in the SBPH transcriptome, and these genes may participate in various cellular processes such as molecular transport, balance maintenance, and degradation of harmful compounds, which may contribute to the adaptation of *Wolbachia* to the SBPH intracellular environment. *Wolbachia* may also provide metabolites needed by the host [67], but information on metabolite supply by *Wolbachia* in SBPH is still lacking.

Apart from the predominant *Wolbachia*, other genera were present at substantially lower proportions. Notably, *Enterobacter* (1.4%) and *Rickettsia* (0.5%) accounted for meaningful proportions of the endosymbiotic community in the VF group. In contrast, within the RSV-viruliferous group, *Burkholderia* emerged as the predominant genus (4.2%) among minor taxa, a finding clearly supported by our PCR validation experiments (Figure S1). Bacteria belonging to the genus *Burkholderia* are widely distributed in the natural environment and are also diverse in their lifestyle, environment, and ecological roles [68,69,70]. Some strains are pathogenic to plants (e.g., *B. gladioli* and *B. glumae*), humans, and other animals [69].; however, most *Burkholderia* species are commonly distributed in soil and roots and have non-pathogenic effects on other organisms [71]. Some of them have established beneficial symbiotic relationships with a variety of eukaryotes, including phytopathogenic and endophytic bacteria, various plants, soil amoebae, and insects [69,70]. To date, in insects, mutualistic symbiotic relationships with *Burkholderia* have been reported for phytophagous stinkbugs (Hemiptera, Pentatomomorpha), ants of the genus *Tetraponera* (Formicidae: Pseudomyrmecinae), Lagrinii beetles (Coleoptera, Tenebrionidae), *Acronicta aceris* (Noctudiae), and *Gossyparia spuria* (Eriococcidae) [72,73,74,75]. To date, the role of *Burkholderia* in SBPH remains unclear. Even if *Burkholderia* potentially influences SBPH-RSV interactions, its overall impact on viral transmission may be limited due to its considerably lower abundance relative to the dominant *Wolbachia* population.

In conclusion, the results of MiSeq sequencing showed that *Wolbachia*, the main endosymbiont, was similar between the two groups, and that *Enterobacter*, *Rickettsia*, and *Burkholderia* made up a small proportion of the remaining endosymbionts, but the expected important endosymbionts such as *Buchnera* and *Hamiltonella* were not found. These are known to be host-specific symbionts (e.g., aphids). These genera may either not naturally occur in SBPHs, or they might exist at concentrations too low in RSV-viruliferous SBPHs to be detected using MiSeq sequencing. In addition, as shown in this study, since *Wolbachia* is a highly dominant endosymbiotic in SBPH in both groups, it opens up the possibility of utilizing *Wolbachia* for suppressing SBPH’s ability to transmit RSV. As mentioned above, *Wolbachia*-induced CI can be used to suppress SBPH populations. However, there have been limited studies on the effects of other identified endosymbionts, including *Wolbachia*, on RSV transmission in SBPH. Although our study provided comprehensive insights into community composition changes in response to RSV infection, further functional validation through targeted experimental manipulation of these endosymbionts is needed to definitively elucidate their roles in SBPH biology and RSV transmission dynamics. Therefore, further studies are warranted. Collectively, our results underline the importance of integrating advanced sequencing techniques and complementary PCR validation to elucidate endosymbiotic dynamics, providing a foundation for developing novel strategies for SBPH control and RSV management.

## 4. Materials and Methods

### 4.1. Insect Rearing

SBPH populations (Non-viruliferous RSV and RSV-viruliferous) used in this study were provided from Kyungpook National University (Sangju, Republic of Korea). RSV-infected rice was also provided, and two-leafed rice was fed every two weeks to maintain SBPH. Confirmation of RSV infection was diagnosed using RSV-specific primers, and the RT-PCR cycles and primers used are described in Section 4.2 and Table S2. SBPH was reared in acrylic cages (40 × 40 × 40, W × D × H, cm) and grown at room temperature under 16:8 (L:D) photoperiod and humidity conditions of 60–70%. Additionally, every two weeks, rice seedlings that had been sprouted for two weeks were newly supplied. The RSV isolate used in this study is the same strain previously sequenced by our research team (NCBI GenBank accession numbers OL438909, OL998468–OL998470).

### 4.2. RNA Extraction and RSV Diagnosis

Twenty SBPHs were randomly selected. RNA was extracted NucleoSpin RNA Plant kit (Machery-Nagel, Düren, Germany) according to the manufacturer’s instructions. RNA concentration and purity were measured using a Nanodrop NP80 (Implen, Munich, Germany). RNA samples were stored at −80 °C before use.

To confirm the RSV, PCR was performed using total RNA and primers for the RNA-dependent RNA polymerase (RdRp) region of RSV. The SuPrimeScript RT-PCR Kit (2X) (Genetbio, Daegeon, Republic of Korea) was used with the following conditions: 50 °C for 30 min, 95 °C for 5 min, followed by 35 cycles of 95 °C for 30 s, 56 °C for 30 s, and 72 °C for 1 min, and a final extension at 72 °C for 5 min. The results were confirmed through electrophoresis. The primers used are described in Table S2.

### 4.3. DNA Extraction, Library Construction and Sequencing

To obtain sufficient high-quality DNA for sequencing with Illumina MiSeq Platform, DNA was extracted from two populations (virus-free and RSV). Each population contained 20 insects (5th-instar nymphs and adults). Genomic DNA was extracted from each SBPH population using the NucleoSpin DNA Insect kit (Machery-Nagel), according to the manufacturer’s instructions. DNA concentration and purity were quantified using a NanoPhotometer^®^ NP80 spectrophotometer (Implen).

Before sequencing, sample quality was measured using the picogreen method with Victor 3 fluorometry. After measurement, the library for the V3-V4 hypervariable region of the bacterial 16s rRNA gene was prepared using the 16S Amplicon PCR Forward Primer = 5′TCGTCGGCAGCGTCAGATGTGTATAAGAGACAGCCTACGGGNGGCWGCAG and 16S Amplicon PCR Reverse Primer = 5′GTCTCGTGGGCTCGGAGATGTGTATAAGAGACAGGACTACHVGGGTATCTAATCC, along with Herculase II Fusion DNA Polymerase (Agilent Technologies, Santa Clara, CA, USA) and the Nextera XT Index V2 Kit (Illumina, San Diego, CA, USA), following the manufacturer’s instructions. The quality of the libraries was also checked using the picogreen method. NGS was performed on the MiSeq platform, producing paired-end 301 bp raw reads, and Library construction and sequencing were conducted by Macrogen (Seoul, Republic of Korea).

### 4.4. 16S Taxonomy Analysis

The raw reads were analyzed using CLC Genomics Workbench software (version 25.0; QIAGEN, Hilden, Germany). Adapters were removed and high-quality reads were separated using the ‘trim read’ tool (quality limit = 0.001, discard below length = 15).

#### 4.4.1. OTU Analysis

The trimmed reads were then used for OTU clustering with the ‘OTU clustering’ tool, analyzed at 99% similarity using the SILVA (SSU 138.1) database. To assess bacterial diversity, indices (Shannon entropy, Phylogenetic diversity, Simpson’s index) were calculated using the ‘alpha diversity’ tool.

#### 4.4.2. ASV Analysis

The trimmed reads were then used for Amplicon Sequence Variants analysis with the ‘Detect Amplicon Sequence Variants and Assign Taxonomies’ tool. The reference database was used the SILVA (SSU 138.1). To assess bacterial diversity, indices (Shannon entropy, Phylogenetic diversity, Simpson’s index) were calculated using the ‘alpha diversity’ tool.

The abundance graphs were created using Excel, and all data analyses were conducted with QIAGEN CLC Genomics Workbench software (version 24.0).

### 4.5. PCR for Endosymbiont Detection

To confirm the presence of endosymbionts in SBPH, genomic DNA extracted from 20 randomly selected individuals (as described in Section 4.3) was used for PCR. PCR amplifications were performed using the SuPrimeScript RT-PCR Kit (2X) (Genetbio), under the identical cycling conditions as described previously (Section 4.2), excluding only the initial reverse transcription step at 50 °C for 30 min. The primers specific for *Rickettsia* and *Burkholderia* were designed to amplify target genes, as listed in Table S2. Actin was used as an internal control.

## Figures and Tables

**Table 1:** Summary of trimmed read counts and alpha diversity indices for bacterial communities of virus-free (VF) and RSV-viruliferous (RSV) SBPH populations analyzed by OTU and ASV methods (99% sequence identity cutoff, equivalent to 1% dissimilarity).

Population	Sample	Total Reads After Trimming	OTUs or ASVs	Total OTUs or ASVs of Population	Shannon Entropy	Phylogenetic Diversity	Simpson’s Index
Nonviruliferous	VF-1-OTU	241,586	54	61	0.62	1.59	0.15
RSV-viruliferous	RSV-1-OTU	327,278	27		0.63	1.11	0.18
Nonviruliferous	VF-1-ASV	241,586	24	34	5.78	1.46	0.98
RSV-viruliferous	RSV-1-ASV	327,278	17		5.72	1.08	0.98

## Data Availability

The data supporting the findings of this study are available from the corresponding author upon reasonable request. The 16S rRNA sequencing data have been deposited in the NCBI Sequence Read Archive (SRA). No additional data were generated that require specific archiving.

## Supplementary Materials

The following supporting information can be downloaded at: https://www.mdpi.com/article/10.3390/v17081074/s1, Figure S1: PCR validation of selected bacterial genera in SBPH populations. Table S1: Raw read counts of 35 bacterial genera from different SBPH populations. Table S2: List of primers used in this study. References [76,77,78] are cited in Supplementary Materials files.
