# Supplementary material for: Comparative Endosymbiont Community Structures of Nonviruliferous and Rice Stripe Virus-Viruliferous Laodelphax striatellus (Hemiptera: Delphacidae) in Korea
Source: Viruses. 2025 Aug 1;17(8):1074. doi: 10.3390/v17081074 (PMC12390706; doi:10.3390/v17081074)
Supplement: Supplementary file 1 [file viruses-17-01074-s001.zip › viruses-3385544-supplementary.pdf]

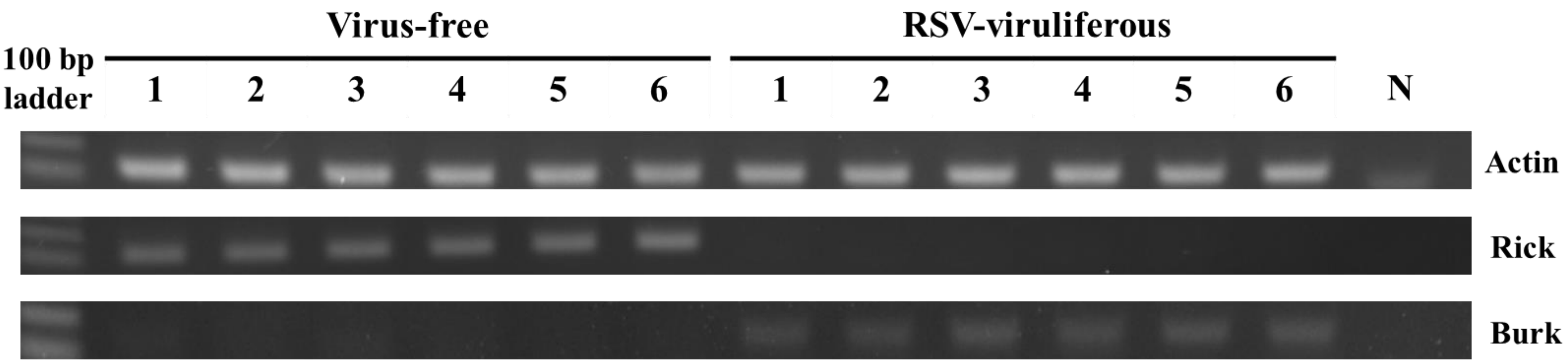

Figure S1. Results of PCR. Virus-free, healthy SBPH; RSV-infected, RSV-viruliferous SBPH; Actin, SBPH's Actin; Rick, Rickettsia; Burk, Burkholderia; N, No template control (water). The primers used are listed in the Table S2.

Table S1. Raw read counts of 35 bacterial genera from different SBPH populations

Genera	Virus-free	RSV-infected
Wolbachia	41655	47599
Enterobacter	645	1
Rickettsia	220	0
Cutibacterium	96	15
Pantoea	88	0
Staphylococcus	55	13
Delftia	49	0
Escherichia-Shigella	45	8
Burkholderia-Caballeronia-Paraburkholderia	44	2115
Cedecea	44	2
Kosakonia	41	0
Taibaiella	22	0
Lawsonella	20	6
Prauserella	18	3
Sphingobacterium	16	0
Acinetobacter	14	0
Corynebacterium	13	2

Allorhizobium-Neorhizobium-Pararhizobium-Rhizobium	10	0
Haemophilus	10	0
Uncultured-1	8	0
Variovorax	8	0
Serratia	8	0
Flavobacterium	7	0
Methylophilus	5	0
Brevundimonas	4	0
Enhydrobacter	4	0
{Unknown Genus} *Corynebacteriaceae*	3	1
Uncultured-2	3	0
Rhodococcus	2	0
Pedobacter	2	0
Brochothrix	2	0
Bradyrhizobium	2	0
Anaerococcus	1	3
Bdellovibrio	0	2
Nevskia	0	31

Table S2. Raw read counts of 35 bacterial genera from different SBPH populations

Target gene	Name	Nucleotide sequence 5′→3′	Size (bp)	Reference	Purpose
LsActin	Actin-F	CGCGATCTGACCGACTACCT	108	This study	PCR
	Actin-R	GTAGCACAGTTTCACCTTGATGTCT			
Burkholderia	BG1F	CCGCGCTGTTCATGAGGGATAA	138	(Kim, et al. 2012)	PCR
	BG1R	CGGGCGGAACGACGGTAAGT			
Rickettsia	PanR8_F	AGCTTGCTTTTGGATCATTTGG	111	(Kato, et al. 2013)	PCR
	PanR8_R	TTCCTTGCCTTTTCATACATCTAGT			
RSV-RdRp	lee-RdRp-F	ATGACGACACCACCTCTCGTTAT	1023	(Lee, et al. 2004)	RSV diagnosis
	lee-RdRp-R	ACTAAGTTTCTGGGAACATAACT			

References

Kato, Cecilia Y, et al. 2013 Assessment of real-time PCR assay for detection of Rickettsia spp. and Rickettsia rickettsii in banked clinical samples. Journal of clinical microbiology 51(1):314-317.

Kim, Byoung Kyu, et al. 2012 Rapid and specific detection of Burkholderia glumae in rice seed by real-time Bio-PCR using species-specific primers based on an rhs family gene. Plant disease 96(4):577-580

Lee, Bong-Choon, et al. 2004 Detection of rice stripe virus using RT-PCR. Research in Plant Disease 10(1):30-33.
